# Supplementary material for: Contrasting maternal and paternal genetic variation of hunter-gatherer groups in Thailand
Source: Sci Rep. 2018 Jan 24;8:1536. doi: 10.1038/s41598-018-20020-0 (PMC5784115; doi:10.1038/s41598-018-20020-0)
Supplement: Supplementary file 1 — Supplementary Information [file 41598_2018_20020_MOESM1_ESM.pdf]

Contrasting maternal and paternal genetic variation of hunter-gatherer groups in Thailand

Wibhu Kutanan[1, 2*], Jatupol Kampuansai[3,4], Piya Changmai[5], Pavel Flegontov[5,6], Roland Schröder[2], Enrico Macholdt[2], Alexander Hübner[2], Daoroong Kangwanpong[3] and Mark Stoneking[2*]

[1]Department of Biology, Faculty of Science, Khon Kaen University, Khon Kaen, 40002, Thailand

[2]Department of Evolutionary Genetics, Max Planck Institute for Evolutionary Anthropology, Leipzig, 04103, Germany

[3]Department of Biology, Faculty of Science, Chiang Mai University, Chiang Mai, 50200, Thailand

[4]Center of Excellence in Bioresources for Agriculture, Industry and Medicine, Chiang Mai University, Chiang Mai, Thailand

[5]Department of Biology and Ecology, Faculty of Science, University of Ostrava, Ostrava, 70103, Czech Republic

[6]Institute of Parasitology, Biology Centre, Czech Academy of Sciences, České Budějovice, 37001, Czech Republic

***Corresponding authors**:

1. Professor Mark Stoneking, Ph.D.

Department of Evolutionary Genetics, Max Planck Institute for Evolutionary Anthropology Deutscher Platz 6, D04103 Leipzig, Germany

Tel: +49 341 3550 502; Fax: +49 341 3550 555; E-mail: stoneking@eva.mpg.de

2. Assistant Professor Wibhu Kutanan, Ph.D.

Department of Biology, Faculty of Science, Khon Kaen University, Mittapap Road, Khon Kaen, 40002, Thailand

Tel: +66 43 202 531; Fax: + 66 43 202 530; Email: wibhu@kku.ac.th

Supplementary Information

Table S1 Details for populations included in the mtDNA comparisons

Citation	Population	Language	Country/Region	Sample size	Code
Present study	Mlabri	Austroasiatic	Thailand	18	MA
Present study	Maniq	Austroasiatic	Thailand	11	MN
Kutanan et al. [33]	Htin	Austroasiatic	Thailand	25	TN1
Kutanan et al. [33]	Htin	Austroasiatic	Thailand	25	TN2
Kutanan et al.[33]	Htin	Austroasiatic	Thailand	25	TN3
Kutanan et al.[33]	Khmu	Austroasiatic	Thailand	25	KA
Kutanan et al.[33]	So	Austroasiatic	Thailand	25	SO
Kutanan et al.[33]	Bru	Austroasiatic	Thailand	24	BU
Kutanan et al.[33]	Seak	Tai-Kadai	Thailand	26	SK
Kutanan et al.[34]	Karen	Sino-Tibetan	Thailand	25	KSK1
Kutanan et al.[34]	Karen	Sino-Tibetan	Thailand	13	KSK2
Kutanan et al. [34]	Karen	Sino-Tibetan	Thailand	24	KPW
Kutanan et al.[34]	Karen	Sino-Tibetan	Thailand	25	KPA
Kutanan et al.[33]	Mon	Austroasiatic	Thailand	25	MO1
Kutanan et al.[33]	Mon	Austroasiatic	Thailand	23	MO2
Kutanan et al.[33]	Mon	Austroasiatic	Thailand	15	MO3
Kutanan et al.[33]	Mon	Austroasiatic	Thailand	25	MO4
Kutanan et al.[33]	Mon	Austroasiatic	Thailand	22	MO5
Kutanan et al.[34]	Mon	Austroasiatic	Thailand	24	MO6
Kutanan et al.[34]	Mon	Austroasiatic	Thailand	25	MO7
Kutanan et al.[33]	Khmer	Austroasiatic	Thailand	19	KH1
Kutanan et al.[33]	Khmer	Austroasiatic	Thailand	25	KH2
Kutanan et al.[33]	Nyahkur	Austroasiatic	Thailand	23	BO
Kutanan et al.[33]	Suay	Austroasiatic	Thailand	25	SU
Kutanan et al.[33]	Blang	Austroasiatic	Thailand	25	BL1
Kutanan et al.[33]	Blang	Austroasiatic	Thailand	25	BL2
Kutanan et al.[33]	Paluang	Austroasiatic	Thailand	25	PL
Kutanan et al.[33]	Lawa	Austroasiatic	Thailand	22	LW1
Kutanan et al.[33]	Lawa	Austroasiatic	Thailand	24	LW2
Kutanan et al.[33]	Lawa	Austroasiatic	Thailand	24	LW3
Kutanan et al. [34]	Khuen	Tai-Kadai	Thailand	25	TKH
Kutanan et al. [34]	Lue	Tai-Kadai	Thailand	25	LU1
Kutanan et al. [34]	Lue	Tai-Kadai	Thailand	23	LU2
Kutanan et al. [34]	Lue	Tai-Kadai	Thailand	25	LU3

Kutanan et al.[34]	Lue	Tai-Kadai	Thailand	16	LU4
Kutanan et al.[33]	Yuan	Tai-Kadai	Thailand	17	YU1
Kutanan et al.[33]	Yuan	Tai-Kadai	Thailand	25	YU2
Kutanan et al.[34]	Yuan	Tai-Kadai	Thailand	25	YU3
Kutanan et al.[34]	Yuan	Tai-Kadai	Thailand	25	YU4
Kutanan et al.[34]	Yuan	Tai-Kadai	Thailand	26	YU5
Kutanan et al.[34]	Yuan	Tai-Kadai	Thailand	25	YU6
Kutanan et al.[34]	CentralThai	Tai-Kadai	Thailand	30	CT1
Kutanan et al.[34]	CentralThai	Tai-Kadai	Thailand	30	CT2
Kutanan et al.[34]	CentralThai	Tai-Kadai	Thailand	30	CT3
Kutanan et al.[34]	CentralThai	Tai-Kadai	Thailand	30	CT4
Kutanan et al.[34]	CentralThai	Tai-Kadai	Thailand	30	CT5
Kutanan et al.[34]	CentralThai	Tai-Kadai	Thailand	29	CT6
Kutanan et al.[34]	CentralThai	Tai-Kadai	Thailand	31	CT7
Kutanan et al.[33]	KhonMueang	Tai-Kadai	Thailand	25	KM1
Kutanan et al.[33]	KhonMueang	Tai-Kadai	Thailand	25	KM2
Kutanan et al.[33]	KhonMueang	Tai-Kadai	Thailand	24	KM3
Kutanan et al.[33]	KhonMueang	Tai-Kadai	Thailand	25	KM4
Kutanan et al.[33]	KhonMueang	Tai-Kadai	Thailand	23	KM5
Kutanan et al.[33]	KhonMueang	Tai-Kadai	Thailand	25	KM6
Kutanan et al.[33]	KhonMueang	Tai-Kadai	Thailand	25	KM7
Kutanan et al.[33]	KhonMueang	Tai-Kadai	Thailand	25	KM8
Kutanan et al.[33]	KhonMueang	Tai-Kadai	Thailand	24	KM9
Kutanan et al.[33]	KhonMueang	Tai-Kadai	Thailand	25	KM10
Kutanan et al.[33]	Shan	Tai-Kadai	Thailand	25	SH
Kutanan et al.[33]	LaoIsan	Tai-Kadai	Thailand	25	IS1
Kutanan et al.[33]	LaoIsan	Tai-Kadai	Thailand	25	IS2
Kutanan et al.[33]	LaoIsan	Tai-Kadai	Thailand	25	IS3
Kutanan et al.[33]	LaoIsan	Tai-Kadai	Thailand	25	IS4
Kutanan et al.[33]	Lao	Tai-Kadai	Laos	25	LA1
Kutanan et al.[33]	Lao	Tai-Kadai	Laos	24	LA2
Kutanan et al.[33]	Phutai	Tai-Kadai	Thailand	25	PT
Kutanan et al.[33]	Kalueng	Tai-Kadai	Thailand	25	KL
Kutanan et al.[33]	Nyaw	Tai-Kadai	Thailand	25	NY
Kutanan et al.[33]	BlackTai	Tai-Kadai	Thailand	25	BT1
Kutanan et al.[33]	BlackTai	Tai-Kadai	Thailand	25	BT2
Kutanan et al.[33]	Phuan	Tai-Kadai	Thailand	25	PU1
Kutanan et al.[33]	Phuan	Tai-Kadai	Thailand	25	PU2
Kutanan et al.[33]	Phuan	Tai-Kadai	Thailand	25	PU3
Kutanan et al.[33]	Phuan	Tai-Kadai	Thailand	25	PU4
Kutanan et al.[33]	Phuan	Tai-Kadai	Thailand	25	PU5
Zhang et al.[63]	Khmer	Austroasiatic	Cambodia	18	KH_C

Zhang et al.[63]	Austroasiatic	Austroasiatic	Cambodia	65	AA_C
Summerer et al.[64]	Barma	Sino-Tibetan	Myanmar	20	BR1
Li et al.[65]	Barma	Sino-Tibetan	Myanmar	73	BR2
Peng et al.[38]	Cham	Austronesian	Vietnam	16	CH
Diroma et al.[66]	Dai	Tai-Kadai	China	56	DA
Zheng et al. [62]	Han	Sino-Tibetan	China	55	HN_S
Zheng et al. [62]	Han	Sino-Tibetan	China	89	HN_N
Zhao et al.[61]	Tibetan	Sino-Tibetan	Tibet and southern China	23	TB
Jinam et al.[13]	Temuan	Austronesian	West Malysia	18	TM
Jinam et al.[13]	Seletar	Austronesian	West Malysia	21	SE
Jinam et al.[13]	Jehai	Austronesian	West Malysia	24	JH
Jinam et al.[13]	Bidayuh	Austronesian	Indonesia	23	BD
Gunnarsdóttir et al.[69]	Semende	Austronesian	Indonesia	35	SMD
Gunnarsdóttir et al.[69]	Besemah	Austronesian	Indonesia	36	BS
Gunnarsdottir et al.[68]	Mamanwa	Austronesian	The Philippines	32	MM
Gunnarsdottir et al.[68]	Manobo	Austronesian	The Philippines	40	MAN
Gunnarsdottir et al.[68]	Surigaonon	Austronesian	The Philippines	25	SR
Delfin et al.[30]	Abaknon	Austronesian	The Philippines	30	AB
Delfin et al.[30]	Aeta_Bataan	Austronesian	The Philippines	21	AEB
Delfin et al.[30]	Bagalot	Austronesian	The Philippines	30	BAG
Delfin et al.[30]	Ibaloi	Austronesian	The Philippines	26	IB
Delfin et al.[30]	Ifugao	Austronesian	The Philippines	29	IF
Delfin et al.[30]	Ivatan	Austronesian	The Philippines	29	IV
Delfin et al.[30]	Kalangoya	Austronesian	The Philippines	26	KAG
Delfin et al.[30]	Kankanaey	Austronesian	The Philippines	30	KAN
Delfin et al.[30]	Maranao	Austronesian	The Philippines	18	MR
Ko et al.[70]	Saisiat	Austronesian	Taiwan	24	SAI
Ko et al.[70]	Atayal	Austronesian	Taiwan	50	ATA
Ko et al.[70]	Tsou	Austronesian	Taiwan	48	TSO
Ko et al.[70]	Bunun	Austronesian	Taiwan	51	BUN
Ko et al.[70]	Puyuma	Austronesian	Taiwan	39	PUY
Ko et al.[70]	Rukai	Austronesian	Taiwan	25	RUK
Ko et al.[70]	Paiwan	Austronesian	Taiwan	50	PAI
Ko et al.[70]	Ami	Austronesian	Taiwan	19	AMI
Ko et al.[70]	Tao	Austronesian	Taiwan	25	TAO
Ko et al.[70]	Hakka	Sino-Tibetan	Taiwan	23	HAK
Ko et al.[70]	Minnan	Sino-Tibetan	Taiwan	25	MIN
Ko et al.[70]	Makatao	Austronesian	Taiwan	50	MAK
Chandrasekar et al.[71]	Malpaharia	Austroasiatic	East India	15	ML
Chandrasekar et al.[71]	Munda	Austroasiatic	East India	31	MUN
Chandrasekar et al.[71]	Andh	Indo-European	Central India	19	AD
Chandrasekar et al.[71]	Dirang Monpa	Sino-Tibetan	Northeast India	30	DR

Chandrasekar et al.[71]	Dongri Bhill	Indo-European	West India	43	DB
Chandrasekar et al.[71]	Gallong	Sino-Tibetan	Northeast India	39	GL
Chandrasekar et al.[71]	Jenu Kuruba	Dravidian	South India	79	JK
Chandrasekar et al.[71]	Kamar	Indo-European	Central India	53	KMR
Chandrasekar et al.[71]	Kathakur	Indo-European	West India	19	KU
Chandrasekar et al.[71]	Kathodi	Indo-European	West India	15	KD
Chandrasekar et al.[71]	Katkari	Indo-European	West India	21	KR
Chandrasekar et al.[71]	Korku	Austroasiatic	Central India	15	KK
Chandrasekar et al.[71]	Lachungpa	Sino-Tibetan	Northeast India	25	LAH
Chandrasekar et al.[71]	Lepcha	Sino-Tibetan	Northeast India	20	LP
Chandrasekar et al.[71]	Mathakur	Indo-European	West India	11	MT
Chandrasekar et al.[71]	Madia	Dravidian	East India	20	MAA
Chandrasekar et al.[71]	Nihal	Indo-European	Central India	28	NI
Chandrasekar et al.[71]	Pauri Bhuiya	Dravidian/Indo-European	Central India	32	PB
Chandrasekar et al.[71]	Shertukpen	Sino-Tibetan	Northeast India	15	ST
Chandrasekar et al.[71]	Sonowal Kachari	Indo-European	Northeast India	19	SOK
Chandrasekar et al.[71]	Toto	Sino-Tibetan	Northeast India	28	TO
Chandrasekar et al.[71]	Wanchoo	Sino-Tibetan	Northeast India	22	WA
Barik et al.[72]	Andaman Islander	N.A.	Andaman Island	10	ADI1
Thangaraj et al.[6]	Andaman Islander	N.A.	Andaman and Nicobarese Island	15	ADI2
Nagle et al.[75]	Aboriginal Australian 1	N.A.	Australia	127	AUS1
Ingman et al. [73]; van Holst Pellekaan et al.[74]	Aboriginal Australian 2	N.A.	Australia	13	AUS2
Macaulay et al. [36]; Dancause et al. [67]	Orang Asli	N.A.	Malaysia	9	MAY
Benton et al. [76]	Maori	Austronesian	New Zealand	20	MAO

Table S2 Details for the populations included in the NRY comparisons

Citation	Population	Language	Country/Region	Sample size
Present study	Mlabri	Austroasiatic	Thailand	10
Present study	Maniq	Austroasiatic	Thailand	4
Kutanan et al. (unpublished data)	Htin	Austroasiatic	Thailand	18
Kutanan et al. (unpublished data)	Htin	Austroasiatic	Thailand	12
Kutanan et al. (unpublished data)	Htin	Austroasiatic	Thailand	17
Kutanan et al. (unpublished data)	Khmu	Austroasiatic	Thailand	18
Kutanan et al. (unpublished data)	Soa	Austroasiatic	Thailand	17
Kutanan et al. (unpublished data)	Bru	Austroasiatic	Thailand	18
Kutanan et al. (unpublished data)	Seak	Tai-Kadai	Thailand	7
Mallick et al.[77]; Karmin et al.[32]	Burmese	Sino-Tibetan	Myanmar	12
Mallick et al.[77]	Papuan	Austronesian	Oceania	12
Karmin et al.[32]	Island Southeast Asian (ISEA) populations	Austronesian	Island Southeast Asia	26
Poznik et al.[78]	Southern Han Chinese	Sino-Tibetan	China	52
Poznik et al.[78]	Northern Han Chinese	Sino-Tibetan	China	44
Poznik et al.[78]	Dai in Xishuangbanna	Tai-Kadai	China	45
Poznik et al.[78]	Kinh	Austroasiatic	Vietnam	46
Karmin et al.[32]	South Asian 1	N.A.	South Asia	25
Mallick et al.[77]	South Asian 2	N.A.	South Asia	31
Poznik et al.[78]	Japanese	Japanese-Ryukyuan	Japan	55
Poznik et al.[78]	Indian Telugu	Dravidian	UK	59
Poznik et al.[78]	Indian Gujarati	Indo-European	Texas, USA	58
Poznik et al.[78]	Bengali	Indo-European	Bangladesh	41
Poznik et al.[78]	Punjabi	Indo-European	Pakistan	48

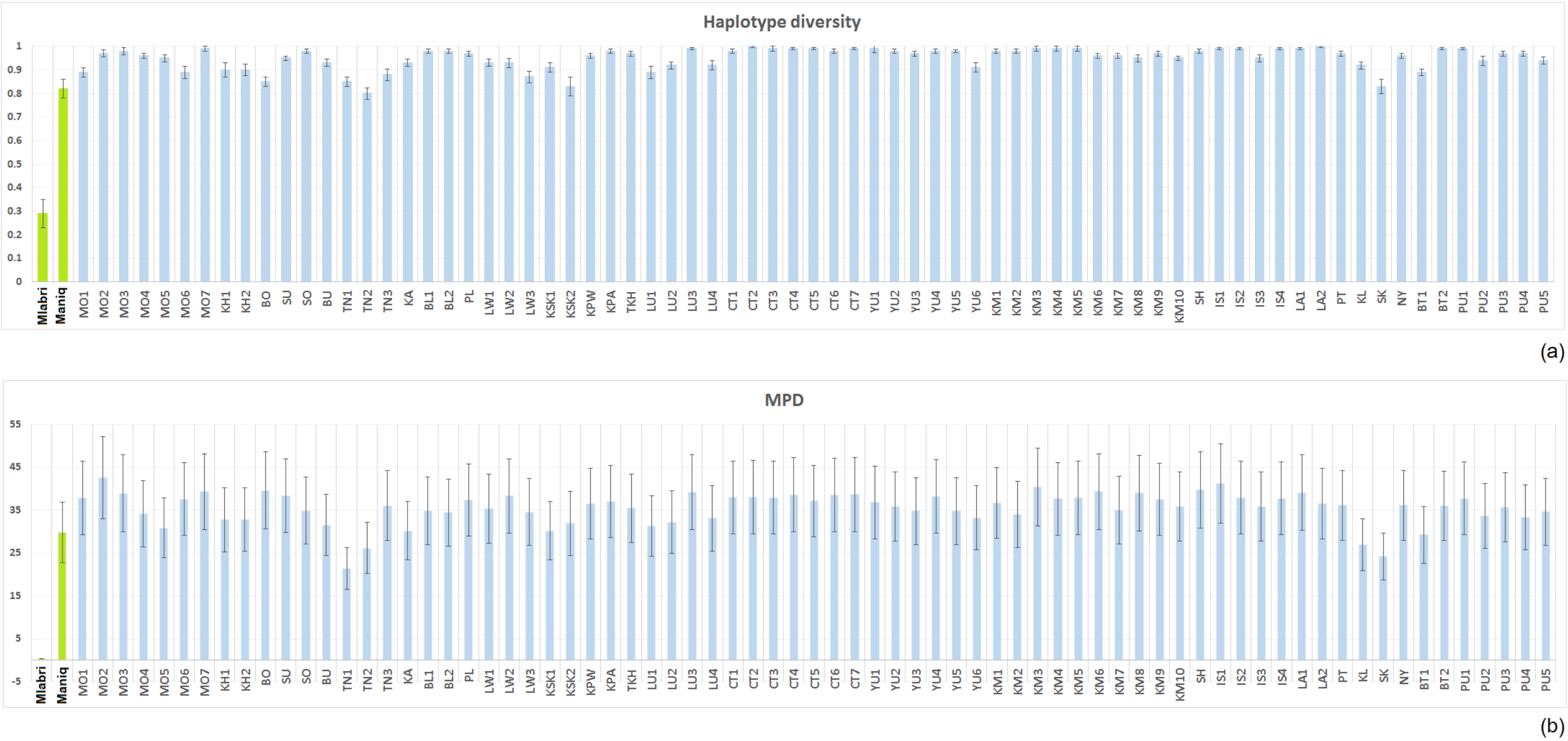

Figure S1 Bar plots showing mtDNA haplotype diversity (a) and mean number of pairwise differences (MPD) (b) in populations from Thailand. See Table S1 for population abbreviations.

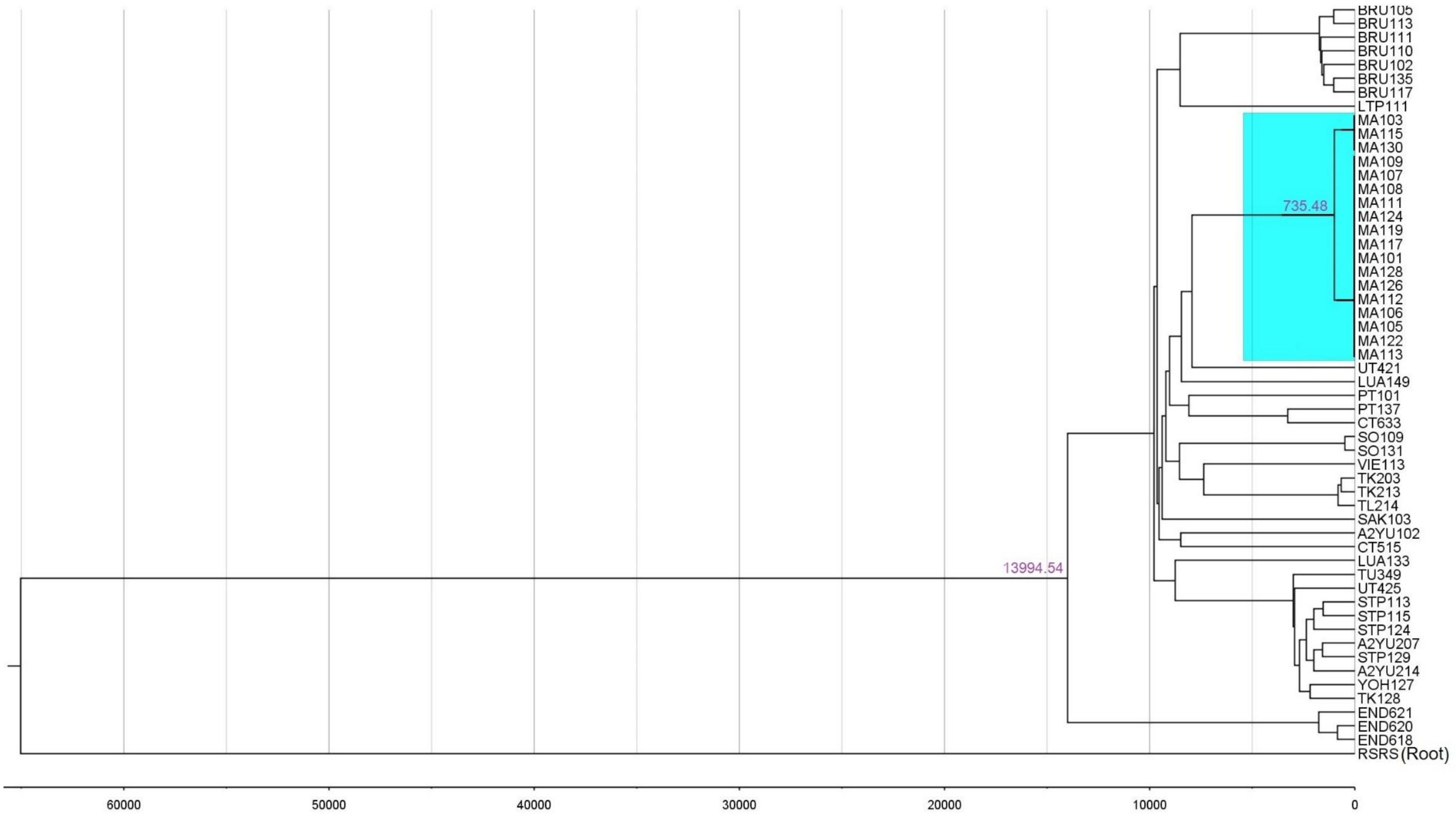

Figure S2 Bayesian maximum clade credibility (MCC) tree of mtDNA sequences belonging to haplogroup B5a1b1 that are found in Thai populations (Kutanan et al.[33,34]). Mlabri sequences are highlighted in blue.

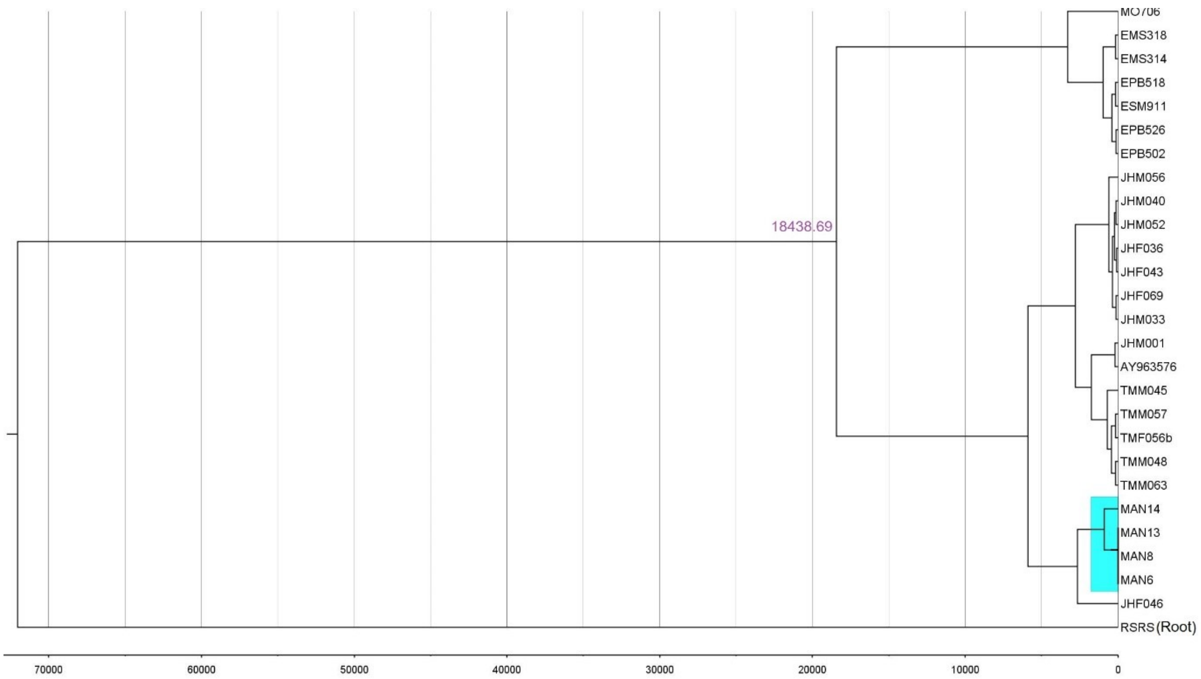
MO706
EMS318
EMS314
EPB518
ESM911
EPB526
EPB502
JHM056
JHM040
JHM052
JHF036
JHF043
JHF069
JHM033
JHM001
AY963576
TMM045
TMM057
TMF056b
TMM048
TMM063
MAN14
MAN13
MAN8
MAN6
JHF046
RSRS (Root)
18438.69
70000
60000
50000
40000
30000
20000
10000
0

(a)

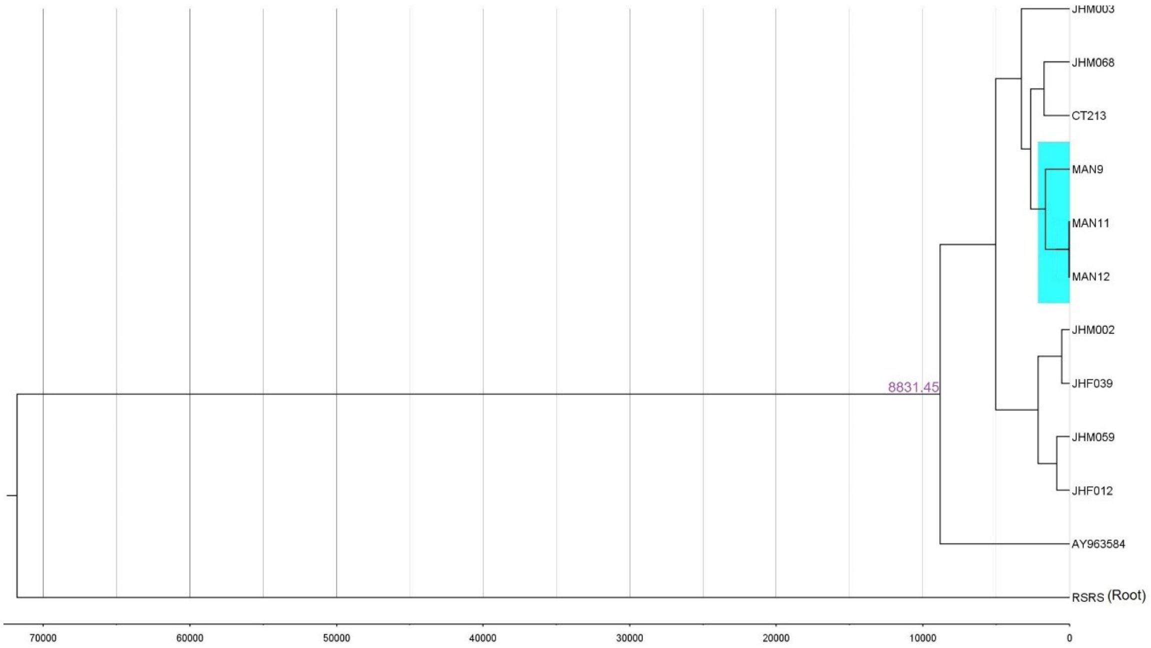
JHM003
JHM068
CT213
MAN9
MAN11
MAN12
JHM002
JHF039
JHM059
JHF012
AY963584
RSRS (Root)
8831.45
70000
60000
50000
40000
30000
20000
10000
0

(b)

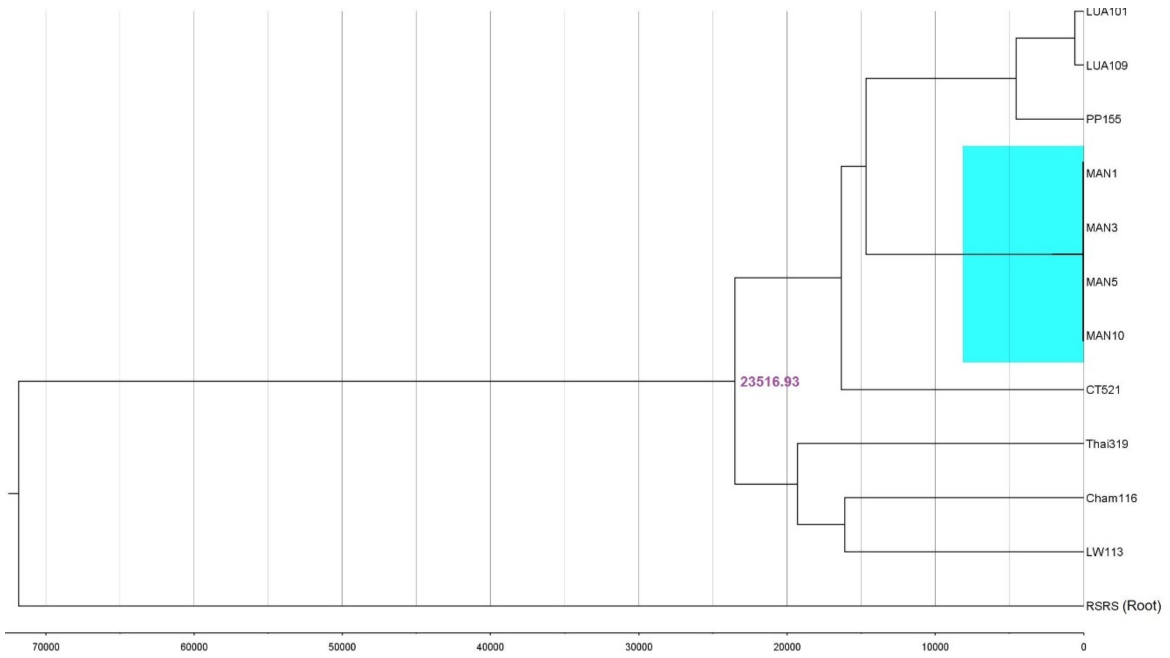

(c)

Figure S3 Bayesian maximum clade credibility (MCC) trees based on mtDNA sequences of Maniq samples and previous samples from Kutanan et al. [33,34], Peng et al.[38], Jinam et al.[11] and an unpublished sample (GenBank number: GU810076.1) of haplogroups M21a (a), R21 (b) and M17 (c). Maniq sequences are highlighted in blue.

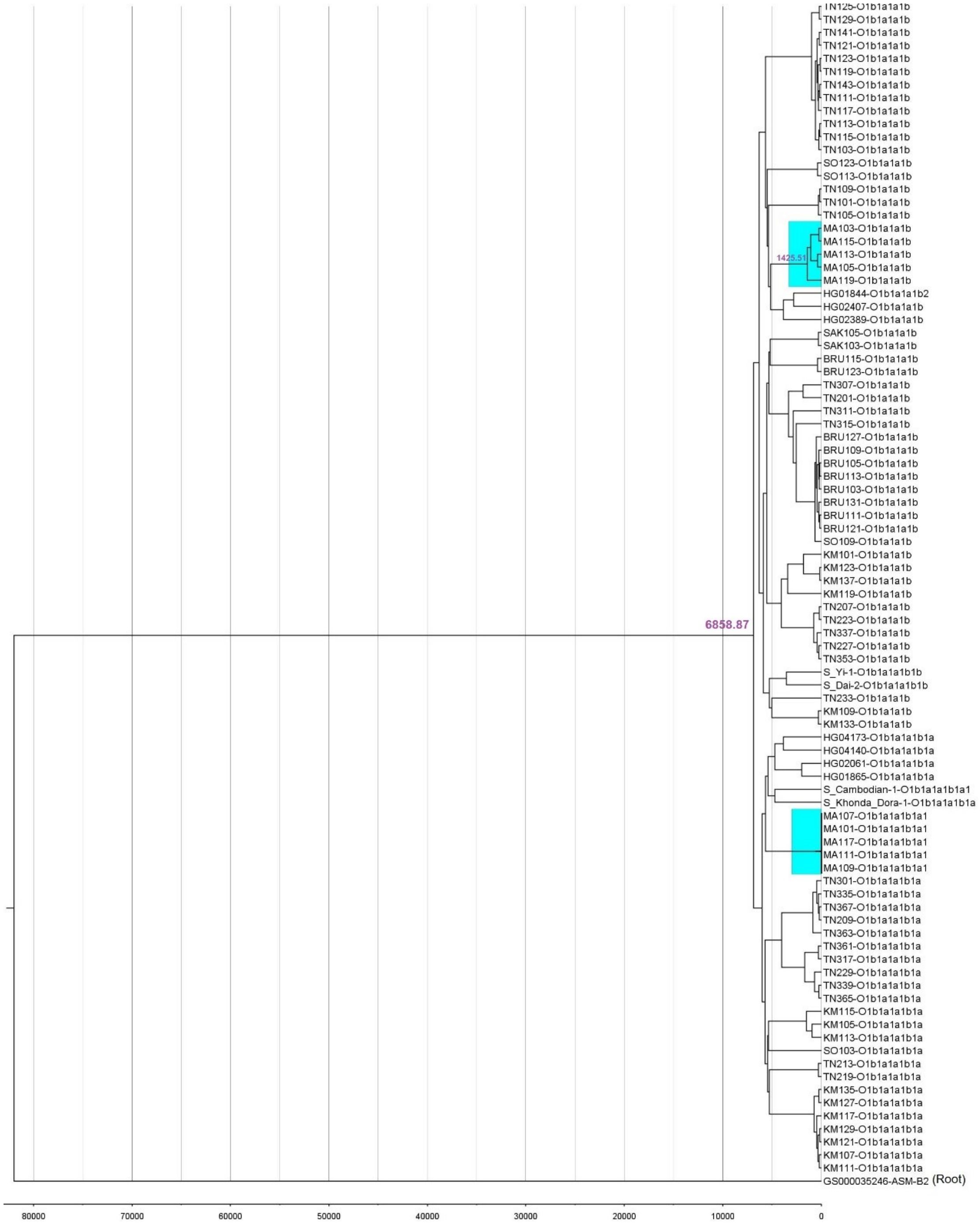

Figure S4 A Bayesian maximum clade credibility (MCC) tree based on NRY sequences of Mlabri and other samples belonging to haplogroup O1b1a1a1b (Kutanan et al. unpublished data; Mallick et al.[77]; Poznik et al.[78]. Mlabri sequences are highlighted in blue.

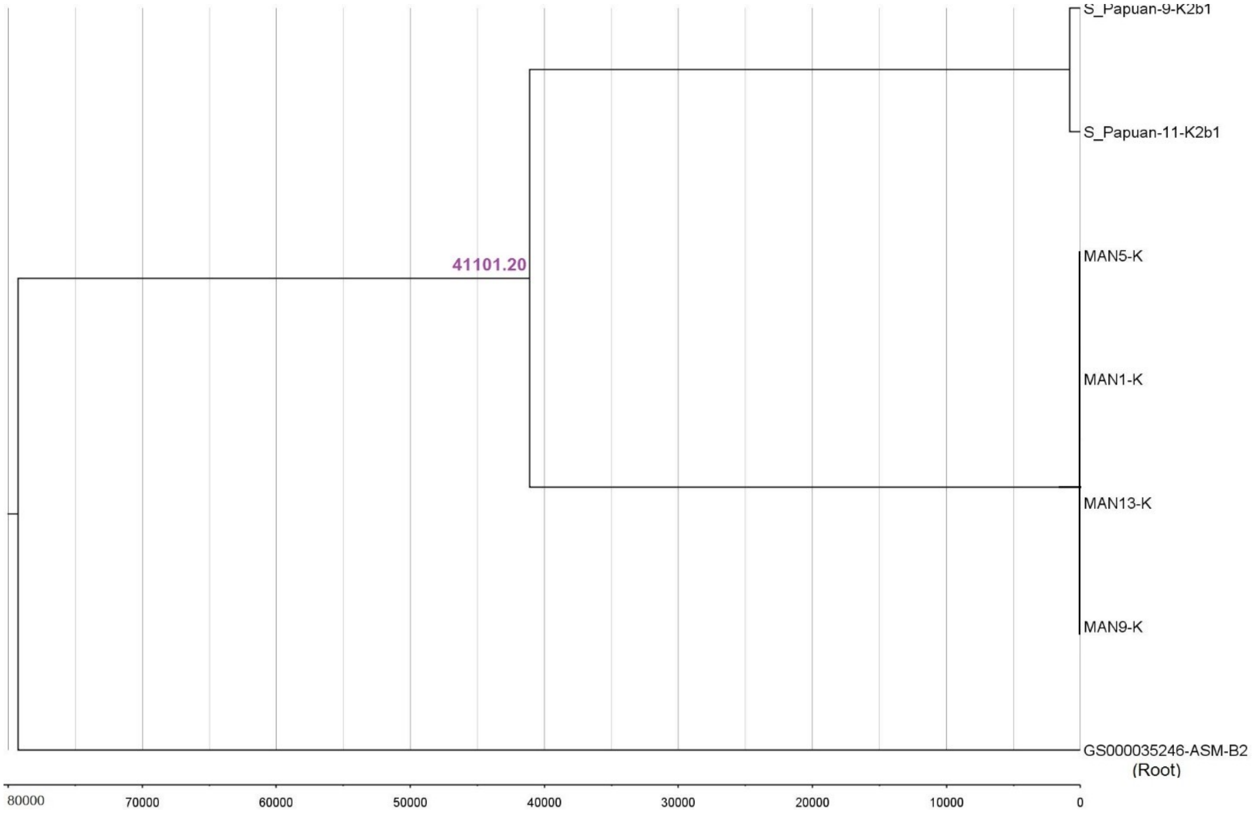

Figure S5 A Bayesian maximum clade credibility (MCC) tree based on NRY sequences of Maniq and Papuans belonging to haplogroup K.
